# Relative Contribution of Arms and Legs in 30 s Fully Tethered Front Crawl Swimming

**DOI:** 10.1155/2015/563206

**Published:** 2015-10-11

**Authors:** Pedro G. Morouço, Daniel A. Marinho, Mikel Izquierdo, Henrique Neiva, Mário C. Marques

**Affiliations:** ^1^Centre for Rapid and Sustainable Product Development, Polytechnic Institute of Leiria, 2430-028 Marinha Grande, Portugal; ^2^Research Centre in Sport, Health and Human Development, 6201-001 Covilhã, Portugal; ^3^Department of Sport Sciences, University of Beira Interior, 6201-001 Covilhã, Portugal; ^4^Department of Health Sciences, Public University of Navarre, Pamplona, 31006 Navarre, Spain

## Abstract

The relative contribution of arm stroke and leg kicking to maximal fully tethered front crawl swimming performance remains to be solved. Twenty-three national level young swimmers (12 male and 11 female) randomly performed 3 bouts of 30 s fully tethered swimming (using the whole body, only the arm stroke, and only the leg kicking). A load-cell system permitted the continuous measurement of the exerted forces, and swimming velocity was calculated from the time taken to complete a 50 m front crawl swim. As expected, with no restrictions swimmers were able to exert higher forces than that using only their arm stroke or leg kicking. Estimated relative contributions of arm stroke and leg kicking were 70.3% versus 29.7% for males and 66.6% versus 33.4% for females, with 15.6% and 13.1% force deficits, respectively. To obtain higher velocities, male swimmers are highly dependent on the maximum forces they can exert with the arm stroke (*r* = 0.77, *P* < 0.01), whereas female swimmers swimming velocity is more related to whole-body mean forces (*r* = 0.81, *P* < 0.01). The obtained results point that leg kicking plays an important role over short duration high intensity bouts and that the used methodology may be useful to identify strength and/or coordination flaws.

## 1. Introduction

The main goal in competitive swimming is to take the shortest time possible to complete an established distance. However, the factors that influence that performance may differ according to the distance to be swum. For instance, in long-distance competitive events swimming technique and race tactics are major factors for success [1]. On the other hand, in sprint events muscular power is crucial [2] since very high speeds are targeted. Therefore, in this latter category muscle force production must be very high to overcome the water resistance and drag [3, 4].

The required force that each swimmer has to apply may be exerted by arms and/or legs, and its assessment may be of great interest for training prescription. Although there have been substantial advances in technology available for swimmers evaluation, the direct measurement of force application during swimming performance remains difficult and challenging [5]. For instance, the use of force plates, usually referenced as the MAD-system, requires the swimmers not to user their legs [6], thus not being able to examine the relative contribution of leg kicking to free swimming. Keeping that in mind, it seems reasonable to use tethered swimming to assess the exerted forces in water, as it has proven to be a valid and reliable methodology [7, 8], with muscular activity [9], oxygen consumption [10], and stroke and physiological responses [11] similar to those seen in free swimming. In fact, tethering a swimmer to a load-cell allows the assessment of each individual force-time curve [12]. This individual evaluation may highlight the ability to effectively use muscular force production in the water, which is more important (and not necessarily related to) compared to assessing strength [13].

On the one hand, both arms and legs are considered the main contributors for force exertion in the water [14, 15], thus being of major importance for performance enhancement over short distance events. Within these segments, their contribution seems to differ, with the leg kicking being commonly considered a factor of secondary importance for front crawl propulsion [16]. It has been stated that its participation enables the achievement of higher velocities by an average value of ~10% [17], but its contribution to overall swimming remains uncertain [14]. In fact, Swaine and colleagues [18] reported a higher importance of leg kicking than that previously assumed, highlighting its contribution to overall propulsion. Their experiments were carried out involving a new swimming training machine to examine the power produced by each limb, which brought new insights into this issue. However, experiments were performed in a dry-land situation being necessary to compare their data with results obtained by experiments carried out in the water.

On the other hand, it has been suggested that combining arms and legs with an appropriate coordination may generate a small amount of additional force exerted in water [18]. Indeed, since early 70s, that front crawl is known to involve highly skilled coordination of the leg kick and arm stroke to enhance utmost forward propulsion [19]. Nevertheless, empirical studies proving this concept are scarce. We do believe that the measurement of forces exerted in the water, restricting the swimmers to use only the arm stroke or the leg kicking, may provide new insights into this issue. Furthermore, it may discern dissimilarities according to gender, as the importance of force and/or technique may vary due to anthropometric characteristics of the swimmer [20]. Indeed, it is uncertain if gender differences in forces measured in tethered swimming during adolescence are similar to the ones described during adulthood [21]. It is known that throughout and up to the end of puberty, males become taller, heavier, and gain more muscle strength than females [22]. Hence, it might be useful to verify if these musculature gains lead to higher capability to effectively apply force in the water.

Research has failed to measure the capability of force exertion in the water using solely arm stroke or leg kicking. Thus, the main purpose of this study was to examine the relative contributions of arm stroke and leg kicking to fully tethered maximal swimming. Likewise, it was intended to examine possible differences in the relative contributions, according to gender, and how these contributions relate to swimming velocity.

## 2. Material and Methods

### 2.1. Subjects

The study included 23 young swimmers with a minimum of 5 years of experience in competitive swimming; main physical and performance characteristics of the subjects are described in Table 1, according to gender. All participants were sprint or middle-distance specialists, participating in national level competitions on a regular basis. Participants were informed about the purpose of the study and any known risks, and parents and coaches gave their consent for inclusion. All procedures followed the 1975 Declaration of Helsinki concerning human research and were approved by the Ethics Committee of the hosting research centre. None of the swimmers suffered from illness or from restrictions that hindered their performances during the experiments. Tests were performed during the competitive period of the spring training cycle to ensure that the participants were in a prime training period.

### 2.2. Experimental Design

**Figure 1:**
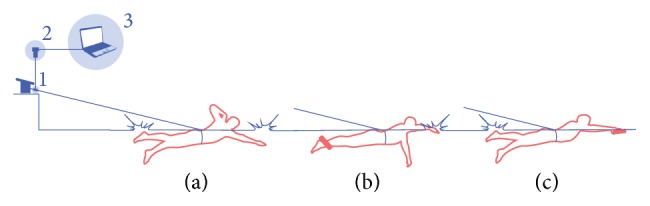
Apparatus used for the fully tethered whole body (a), arm stroke (b), and leg kicking (c) swimming tests: 1 = load-cell; 2 = ergometer data acquisition system; 3 = personal computer.

The swimmers completed a 1000 m standardized warm up (400 m swim, 100 m pull, 100 m kick, 4 × 50 m at increasing speed, and 200 m easy swim) before performing 3 × 30 s maximal intensity fully tethered front crawl swimming. The tests were performed in a randomized order and were separated by a minimum of 20 min active recovery. For 1 test, no constrains were applied so that participants could use their whole body (cf.  Figure 1(a)). For the other 2 tests, floating devices (pull buoys) were used to restrict the movements of legs or arms (cf. Figures 1(b) and 1(c), resp.). Visual inspection by the scientific personnel was made to verify if the swimmers were able to keep their streamlines for each condition; if not, the test was repeated. In addition, the participants had their ankles fastened together to prevent them from performing leg kicking when assessing forces for the arm stroke test (Figure 1(b)). On the subsequent day, swimmers performed 2 maximal 50 m front crawl swimming bouts with an in-water start, to diminish the effect of starts and glide. All experimental testing was performed in a 50 m indoor swimming pool with a water temperature range of 25.5–27°C.

### 2.3. Experimental Measurements

A 3.5 m inextensible steel cable was used to attach the participants to the starting block. Between the cable and the starting block a load-cell was used to measure forces exerted by the swimmers. Equipment provided a recording rate of 100 Hz and a maximum capacity of 4905 N. The load-cell was connected to a Globus Ergometer data acquisition system (Globus, Italy), which exported the data in ASCII format to a PC. Preceding the starting signal, swimmers adopted a horizontal position with the cable fully extended and performed for 5 s at low intensity. Data collection only started after the completion of the first maximum cycle to avoid the inertial effect of the cable extension, usually observed during the first upper limb action. The participants were told to follow the breathing pattern they would normally apply during a 50 m freestyle event and were verbally encouraged throughout the tests to maintain maximal effort during the 30 s. The end of the tests was set through an acoustic signal emitted by the scientific personnel.

### 2.4. Data Analysis

ASCII data files were exported to signal processing software (AcqKnowledge v.3.7, Biopac Systems, Santa Barbara, USA) and filtered through a 4.4–4.8-Hz cut-off low-pass fourth-order Butterworth filter. The cut-off value was chosen according to residual analysis (residual error versus cut-off frequency). As the force vector in the tethered system presented a small angle (5.7°), data was corrected computing the horizontal component of force. Individual force-time curves were assessed to estimate maximum and mean values. Swimming velocity was calculated from the 50 m free swim best time.

### 2.5. Statistical Analysis

Normality and homoscedasticity assumptions were checked by Shapiro-Wilk and Levene tests, respectively. The significance of differences between genders was evaluated with an independent samples *t*-test. Repeated measures analyses (ANOVA, with Bonferroni post hoc test) and Pearson product-moment correlation coefficients (*r*) were calculated among the tests, controlling the swimmers gender. All statistical procedures were performed using SPSS 20.0 (Chicago, IL, USA). The level of statistical significance was set at 95% (*P* < 0.05).

## 3. Results

**Figure 2:**
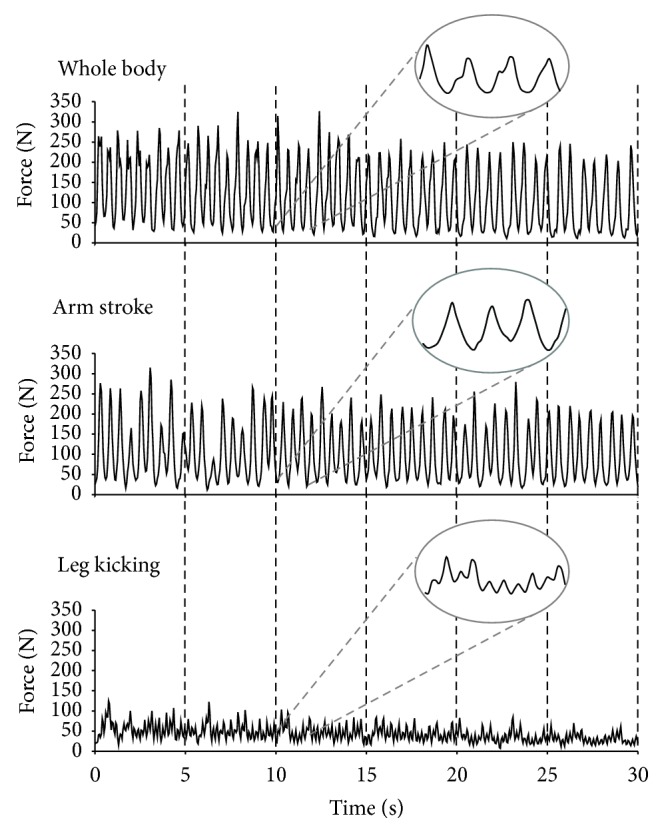
Illustrative typical force profiles in a 30 s tethered whole body, arm stroke, and leg kicking swimming tests.

Illustrative force-time curves for the 3 tests are shown in Figure 2. Different profiles can be perceived as the restriction of legs or arms affected swimmers capability to exert force in the water. When the swimmer increases the force exertion, an upward trace arises and, on the other hand, when the force exertion decreases a downward trace occurs.

**Figure 3:**
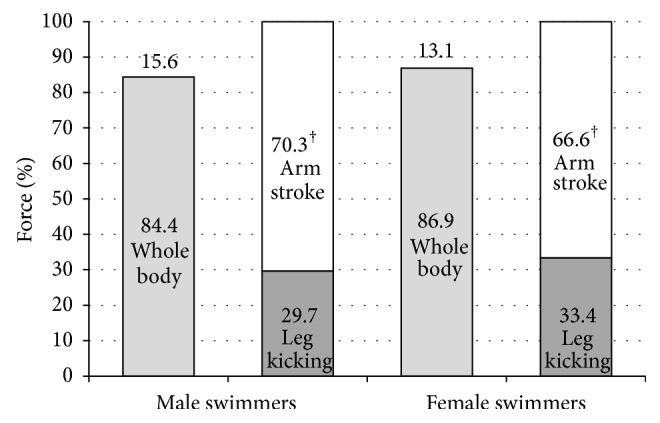
Relative contribution (%) of arms and legs in fully tethered front crawl swimming, according to gender. ^†^ *P* < 0.01 compared with the whole-body conditions. Numbers in the columns represent mean values.

In Table 2, the maximum and mean forces obtained for each condition are displayed, according to gender. With no restrictions, swimmers were able to exert higher forces than using only their arm stroke or leg kicking (*P* < 0.001). The sum of arm stroke and leg kicking mean forces was higher than the whole-body mean force for all subjects, except for two female swimmers. Estimated relative contributions of arm stroke and leg kicking were 70.3% versus 29.7% for males and 66.6% versus 33.4% for females. Considering the sum of arm stroke and leg kicking mean forces as 100%, the forces exerted using the whole body were 84.4 ± 6.8% for males and 86.9 ± 9.9% for females. This corresponded to a force deficit of 15.6% and 13.1%, respectively (cf.  Figure 3).

In the males group, the ones that obtained higher maximum forces were not the ones that produced higher mean forces (*r* = 0.46, *P* > 0.05), whereas in the females group there was a significant association between the maximum and mean forces (*r* = 0.76, *P* < 0.01). For both genders, forces exerted using the whole body were positively related to the forces obtained through arm stroke (*r* = 0.7, *P* < 0.05) and leg kicking (*r* = 0.8, *P* < 0.05).

Male swimmers performed higher swimming velocities than their female counterparts (1.71 ± 0.05 versus 1.53 ± 0.11 m/s, *P* < 0.01). The velocities presented higher correlations with the arm stroke maximum force for males (*r* = 0.77, *P* < 0.01) and with the whole-body mean force for females (*r* = 0.81, *P* < 0.01).

## 4. Discussion

To the best of our knowledge, few studies have aimed to analyze the importance of leg kicking in front crawl swimming [16, 17]. Thus, this study aimed to examine the relative contribution of arm stroke and leg kicking to force production in front crawl fully tethered swimming. The major findings of this study were that (i) leg kicking played a high relative contribution (~31%) and (ii) male swimmers achieved higher swimmer velocities due to the high force exertion with their arm stroke, whereas female swimmers swimming velocities were more related to whole-body mean forces. Additionally, the obtained results permitted the estimation of a force deficit, which may be a useful procedure to identify lack of strength and/or coordination.

For all swimmers tested, maximum and mean forces were higher using their whole body, followed by using only the arm stroke and followed by the leg kicking. Even though these are pioneering results, they are in accordance with the expectations, as propulsive capacity decreases in each situation. Previous studies pointed out that the arm stroke generates 90% of the total propulsive thrust in sprint freestyle [16, 17]. Using the 20 m swimming speeds with no constraints and using only the arm stroke, Deschodt and colleagues [17] indirectly reported that 10% higher speed was achieved when using the leg kicking. These authors did not normalize the data to a leg kicking condition and yet their percentage was lower than the one obtained in the present study, with lower level swimmers. On the other hand, a recent study [18] reported a 62.7 ± 5.1% and 37.3 ± 4.1% mean contribution of arms and legs, respectively, using a dry-land novel machine. Current higher percentages undoubtedly put in question the statements that swimming propulsion is almost entirely due to arms and trunk [23], at least over short duration and high intensity bouts. A relative contribution of ~31% (cf.  Figure 3) may reinforce the suggestion that a much greater proportion of the force exerted in water to increase swimming speed may be attributable to the legs than previously thought [14]. The small bias between the experiment of Swaine and colleagues [18] and the present study may be due to the environment conditions, as the former was performed in dry land.

In front crawl swimming, the upper and lower limbs perform alternated movements to produce propulsive actions. Except for 2 female swimmers, the sum of forces exerted by arm stroke and leg kicking was higher than using the whole body. These results suggest that combining upper and lower limbs with an appropriate coordination may generate a small amount of additional force exerted in water. In doing so, a powerful leg kick may be almost as important as a powerful arm stroke in swimming, even though the leg kick contributes much less to propulsion [14]. The used methodology allowed identifying swimmers that were not able to achieve as much force with no constrains, as with the sum of arms and legs separately. These differences can be considered as a force deficit (which was similar among genders), providing the diagnosis of coordination flaws. In fact, low values may represent situations where strength development of arms and legs might not lead to a gain in performance, as the necessary coordination would be deficient [17]. Interestingly, Ogita et al. [24] reported that the total energy production during swimming was lower than the sum of energy production during separately measured arm stroke and leg kicking swimming. It seems, therefore, that the potentials of both the anaerobic and aerobic energy releasing processes in the muscle groups involved in arm and leg actions cannot be fully reached during free swimming [24].

Regarding the gender comparison, male swimmers obtained both higher forces and velocity than female swimmers. These gender biases may be related to anthropometrical differences (cf. Table 1), but similarities between relative contribution and force deficit should be examined. First, in order to avoid errors due to the stationary swimming, current data was obtained on competitive swimmers already familiarized with fully tethered swimming methodology, as recommended [8]. Second, it is well described in the literature that several factors influence swimming performance. For instance, both head [25] and thumb [26] positioning have proven to be influencing factors. To overcome the limitation of not controlling every variable, we assumed that swimmers would maintain similar positioning among tests. Third, apart from being taller, heavier, and with longer limbs, as is common in postpubescent stages [27, 28], the higher forces that male swimmers obtained may induce that they had higher muscle strength levels than females, which is in accordance with previous findings that point to a strength differential after puberty [28, 29]. Furthermore, our results suggest that those swimmers with higher strength levels are also those with higher swimming speed, being partially related to a greater capacity to apply propulsive force to water. Nevertheless, the relative contributions of arm stroke and leg kicking were parallel among genders. Thus, the used procedures can be a novel approach to assess the forces exerted in each condition, in order to evaluate the relative contributions of arm stroke and leg kicking.

Relationships between front crawl tethered swimming and swimming performance have been previously studied [30–32], mostly with heterogeneous samples indifferently analyzing male and female swimmers [12, 16]. Studies showed that the stroke force that swimmers could generate was moderately to highly related to swimming speed in sprint distance efforts. However, coupling results of heterogeneous samples can discredit the results [33], and the analyses per gender might clarify performance differences in adolescent swimmers. Indeed, proper scrutiny is to be recommended, since associations of maximum or mean forces differ. It was confirmed that maximum force is a better estimator for swimming performance in male swimmers, while mean values are more appropriate for female swimmers. Differences were also noticeable for arm stroke and leg kicking experiments. In fact, the musculature of the upper body seems highly correlated with sprint performance in male swimmers. On the contrary, in female participants, whole body plays a major role over short-distance swimming performance. As remarked earlier, differences in musculature and strength become notorious at these ages [27] and should be considered when prescribing strength training.

## 5. Conclusions

Swimming coaches are aware that the evaluation of their swimmers should be specific to the nature of the sport. Therefore, it is essential that the chosen apparatus strongly replicates the movement patterns (if possible with the same musculature demands) employed in real training and competition situations. In summary, both arm stroke and leg kicking were a two-independent factor that took a major role in sprint distances, so that leg kicking should not be neglected in sprint swimmers training. In male swimmers, upper limbs musculature was able to reach very high values of exerted forces strongly influencing swimming performance. For female swimmers, the ability to keep force production during the 30 s, that is, mean forces, was more related to swimming performance. The used methodology may be useful to diagnose the lack of strength or coordination.

## Figures and Tables

**Table 1:** Main physical and performance characteristics of the subjects, according to gender.

	Males (*n* = 12)	Females (*n* = 11)
Age (years)	15.2 ± 0.9	15.7 ± 1.4
Height (m)	1.73 ± 0.07^*^	1.61 ± 0.06
Upper limb length (cm)	64.8 ± 2.1^†^	58.1 ± 3.6
Lower limb length (cm)	82.5 ± 3.6^†^	75.8 ± 4.4
Body mass (kg)	61.8 ± 7.1^†^	55.7 ± 5.8
Body fat (%)	11.7 ± 3.1^†^	23.7 ± 3.6
Personal best 100 m freestyle (s)	59.5 ± 2.0^†^	67.1 ± 5.9

Values are mean ± SD; ∗ and † significantly higher than females (*P* < 0.05 and *P* < 0.01, resp.).

**Table 2:** Data collected from the 30 s fully tethered front crawl swimming tests, according to gender.

	Males (*n* = 12)	Females (*n* = 11)
Maximum force (N)		
Whole body	325.4 ± 27.8^†^	222.3 ± 61.8
Arm stroke	243.7 ± 27.7^†^	168.5 ± 36.2
Leg kicking	100.1 ± 28.2^†^	72.0 ± 9.4
Mean force (N)		
Whole body	98.8 ± 13.7^†^	74.0 ± 12.4
Arm stroke	82.5 ± 12.0^†^	56.9 ± 8.7
Leg kicking	35.1 ± 7.6^†^	28.4 ± 4.6

Values are mean ± SD; ^†^significantly higher than the females (*P* < 0.01).
